# Urb-RIP – An Adaptable and Efficient Approach for Immunoprecipitation of RNAs and Associated RNAs/Proteins

**DOI:** 10.1371/journal.pone.0167877

**Published:** 2016-12-08

**Authors:** Kyle A. Cottrell, Sergej Djuranovic

**Affiliations:** Department of Cell Biology and Physiology, Washington University School of Medicine, St. Louis, Missouri, United States of America; John Curtin School of Medical Research, AUSTRALIA

## Abstract

Post-transcriptional regulation of gene expression is an important process that is mediated by interactions between mRNAs and RNA binding proteins (RBP), non-coding RNAs (ncRNA) or ribonucleoproteins (RNP). Key to the study of post-transcriptional regulation of mRNAs and the function of ncRNAs such as long non-coding RNAs (lncRNAs) is an understanding of what factors are interacting with these transcripts. While several techniques exist for the enrichment of a transcript whether it is an mRNA or an ncRNA, many of these techniques are cumbersome or limited in their application. Here we present a novel method for the immunoprecipitation of mRNAs and ncRNAs, Urb—RNA immunoprecipitation (Urb-RIP). This method employs the RRM1 domain of the “resurrected” snRNA-binding protein Urb to enrich messages containing a stem-loop tag. Unlike techniques which employ the MS2 protein, which require large repeats of the MS2 binding element, Urb-RIP requires only one stem-loop. This method routinely provides over ~100-fold enrichment of tagged messages. Using this technique we have shown enrichment of tagged mRNAs and lncRNAs as well as miRNAs and RNA-binding proteins bound to those messages. We have confirmed, using Urb-RIP, interaction between RNA PolIII transcribed lncRNA BC200 and polyA binding protein.

## Introduction

Regulation of gene expression at the post-transcriptional level is a complex process involving many *trans* factors such as RNA-binding proteins (RBPs), non-coding RNA (ncRNA), and ribonucleoproteins (RNPs) [1, 2]. In order to fully understand this process for any given RNA it is essential that we know what factors are bound to the transcript. This knowledge will prove useful in designing therapies that target *trans* factors or the RNA itself. While there exist many molecular techniques for purification of RNAs of interest [3–18] and *in silico* tools for identification of RNA binding protein (RBP) or ncRNA binding sites on your RNA of interest [19–24], many of these tools have limitations in their applicability, efficiency or false positive rate. Current techniques for RNA purification fall into one of three classes: RBP-mediated [3–10], aptamer and oligonucleotide-mediated [11–18], or direct purification of biotinylated RNA [25, 26]. While each of these techniques has been used successfully, they often require unique experimental designs that make them potentially less adaptable and more time consuming.

Pulldown of RNA of interest using aptamers or oligonucleotides relies on base pairing of an biotinylated-oligonucleotide to an RNA of interest or binding of a structure inserted into an RNA of interest to compound, usually a metabolite. While both techniques have been used successfully they both suffer from the same difficulty, RNA structure. Folding of the RNA can disrupt the formation of the aptamer or occlude the binding site of an oligonucleotide. For pulldown with oligonucleotides this can be abrogated by tiling across the entire RNA with multiple oligonucleotides; however this increases the chance of pairing with other RNAs beside the RNA of interest.

Direct purification of a biotinylated RNA involves *in vitro* synthesis of an RNA of interest and tagging with biotin, this is usually done with a biotinylated cap or 5’ nucleotide. The biotinylated RNA is then incubated with a cell lysate and subsequently precipitated with a streptavidin matrix. The biggest drawback of this technique is that the RNA is introduced to a lysate as opposed to being transcribed within the cell as normal. It is well appreciated that numerous proteins bind to RNAs concurrent with transcription or splicing. These interactions may not occur when an *in vitro* transcribed RNA is incubated with a cell lysate, potentially leading to false negative results.

Likely the most common technique for affinity based RNA-purification is RNA-immunoprecipitation (RIP) using the bacteriophage MS2-coat protein. This approach uses an epitope tagged MS2-protein to enrich an RNA of interest containing the MS2 hairpin. While this technique has been widely and successfully used [3–8] it is not without its pitfalls. The main pitfall being a lack of efficiency; RNA of interest are routinely tagged with multiple MS2-hairpins often up to two dozen [3–8]. The addition of a large number of MS2-hairpins adds a significant amount of mass to the RNA of interest and can result in relatively poor enrichment, less than one order of magnitude [3].

Here we report a new method for targeted RNA pull-down that is both efficient and highly adaptable. Our approach, which we have named Urb—RNA immunoprecipitation (Urb-RIP), utilizes the RNA recognition motif 1 (RRM1) domain of the “resurrected” snRNA-binding protein Urb to enrich transcripts containing a stem-loop tag. The RRM1 domain of Urb binds stem-loop II (SLII) of the U1-snRNA and SLIV of the U2-snRNA with high affinity [27]. Urb-RIP uses a single SLII-tag to allow binding of Urb-RRM1 to an RNA of interest. Prior to cell lysis we employ crosslinking by UV irradiation to produce RNA-protein crosslinks between Urb and the tagged RNA or other proteins bound to the RNA much like CLIP techniques [28, 29]. Following immunoprecipitation it is possible to specifically elute RNA or protein bound to the RNA of interest. We have validated Urb-RIP using transcripts generated by RNA polymerase II and III. Pull-down of mRNAs was highly efficient and provided enrichment of RNA binding proteins bound to the message. Using Urb-RIP pull-down of a miRNA-targeted reporter we have enriched for the miRNA which targets the tagged mRNA as well as Argonaute protein, part of the miRNA-induced silencing complex (miRISC). Finally, we confirmed the binding of polyA-binding protein (PABP) to the RNA PolIII transcribed lncRNA BC200 using Urb-RIP.

## Materials and Methods

### Construction of 2HA-Urb and SLII-tagged RNA constructs

All primers used for cloning can be found in S1 Table. The RRM1 domain of Urb was a kind gift from the laboratory of Kathleen Hall. Two rounds of PCR were performed with first 2HA-RRM1-URB forward-1 and then 2HA-RRM1-URB forward-2 each with 2HA-RRM1-URB reverse to add 2x HA tags followed by a TEV protease site to URB-RRM1. 2HA-TEV-URB-RRM1 was then cloned into pENTR-D-TOPO (Invitrogen). Site-directed mutagenesis was performed using the 2HA-URB NarI mutagenesis primers to introduce a *NarI* restriction site in between the coding region for the TEV protease and N-terminus of URB-RRM1. The pENTR-2HA-TEV-URB-RRM1 plasmid containing the inserted *NarI* site was then digested with *NarI* and ligation was performed to insert a single FLAG tag using the FLAG oligonucleotides 1 and 2. LR-Clonase II (Invitrogen) was used to transfer the pENTR-2HA-TEV-FLAG-URB-RRM1 insert to the destination plasmid pT-RexDEST31 (Invitrogen), making pT-REx-2HA-TEV-FLAG-URB-RRM1 (referred to above in the text as pT-REx-2HA-URB). The pENTR-2HA-TEV-FLAG-URB-RRM1 plasmid was also recombined with pCDNA5-Frt-TO (Invitrogen) to make a pCDNA5-2HA-URB.

To facilitate SLII-tagging of mRNAs a destination vector was constructed that would place a SLII-tag in the 3’UTR. The SLII-tag was inserted into the 3’UTR of pcDNA-DEST40 to make pcDNA-DEST40-SLII. pcDNA-DEST40 was digested with *SacII* and ligation was performed to insert the SLII-tag using the SLII Tag *SacII* oligonucleotides 1 and 2

LR-Clonase II (Invitrogen) was used to transfer the pENTR-mCherry insert to the destination plasmid pcDNA-DEST40 or pcDNA-DEST40-SLII, making pcDNA-mCh and pcDNA-mCh-SLII.

The transcript sequence for the lncRNA-BC200 was amplified from HEK 293 genomic DNA purified using DNeasy Blood & Tissue Kit (Qiagen) by the BC200 forward and BC200 reverse (with PolII terminator) primers. The PCR product was digested with *SpeI* and *XbaI* and ligated into pSM2 vector (Addgene) digested with the same restriction enzymes. The SLII-tag with *XbaI/SpeI* overhangs, SLII Tag *SpeI* oligonucleotides 1 and 2, was ligated into the pSM2-BC200 plasmid digested with *SpeI*. The inserts from pSM2-BC200 and pSM2-SLII-BC200 were recombined into pcDNA-DEST40ΔCMV using LR-Clonase II (Invitrogen) to make pcDNAΔ-BC200 and pcDNAΔ-SLII-BC200. pcDNA-DEST40ΔCMV was constructed by digest to remove the CMV promoter with *SpeI* (NEB) and *SacI* (NEB) followed by ligation with the CMV deletion oligonucleotides 1 and 2.

A region of pAWH-Rluc-*let-7*-A114-N40-HhR [30] containing eight *let-7* binding sites was amplified using the pAWH *let-7* sites forward and reverse primers. This PCR product was gel purified and phosphorylated using T4-PNK (NEB). This product was then ligated into the pcDNA-mCh and pcDNA-mCh-SLII plasmids that had been digested with *PmeI* and dephosphorylated with Antarctic phosphatase (NEB).

To make EGFP-FLAG-AGO2, EGFP was PCR amplified using the EGFP forward and reverse-overlap primers and Ago2 was amplified using the Ago2 forward-overlap and reverse primers. The PCR products were stitched together using overlap PCR and cloned into pENTR-D-TOPO. LR-Clonase II (Invitrogen) was then used to transfer the EGFP-FLAG-Ago2 cassette into pcDNA-DEST40 making pcDNA-EGFP-FLAG-Ago2.

### Cell culture and transfection

T-REx^™^-293 cells and Flp-In T-REx^™^-293 (Invitrogen) were grown in DMEM (Gibco) supplemented with 10% heat-inactivated FBS (Gibco), 1x Penicilin streptomycin and glutamine (Gibco) and 1x MEM Non-Essential Amino Acids (Gibco). T-REx^™^-293 were kept under selection with 5 μg/mL blasticidin. Transfection was performed using Xtreme Gene 9 (Roche) per manufacturer’s recommendations. The plasmid was transfected at a ratio of 1 μg per 2 μL of transfection reagent. For a 10 cm dish 8 μg of plasmid was used. Two stable cell lines were made for expression of 2HA-Urb. The pT-REx^™^-2HA-Urb plasmid was transfected into T-REx^™^-293 cells and the cells were selected with 0.5 mg/mL Geneticin (Invitrogen) to produce a stable cell line. This cell line (T-REx^™^-293 -2HA-Urb) was maintained in 5 μg/mL blasticidin and 0.5 mg/mL geneticin. The pCDNA5-2HA-Urb plasmid was co-transfected with the Flippase expressing plasmid pOG-44 (Invitrogen) into Flp-In T-REx^™^-293 and the cells were selected with 0.1 mg/mL hygromycin to produce a stable cell line. This cell line (Flp-In T-REx^™^-293-2HA-Urb) was maintained in 5 μg/mL blasticidin and 0.1 mg/mL hyrgomycin. These cell lines were used interchangeably with minimal differences in Urb-RIP efficiency (data not shown).

### Crosslinking and immunoprecipitation of SLII-tagged RNA (Urb-RIP)

A detailed protocol for the Urb-RIP method and the recipes for all buffers listed below can be found in the Supplemental Methods (S1 Text), this protocol has been adapted from previous CLIP protocols [28, 29]. Prior to performing Urb-RIP one 10 cm plate of T-REx-293 -2HA-Urb or Flp-In T-REx-293-2HA-Urb cells was transfected with a tagged RNA of interest or an untagged control as described above. Four hours later the media was removed and 2HA-Urb expression was induced by addition of fresh media containing 2 μg/mL doxycycline. The next day the cells were transferred into a single 15 cm dish in media containing doxycycline as before. The following day the cells were washed briefly with cold PBS prior to UV-irradiation at 400 mJ/cm^2^ using a Stratalinker 1800. The cells were suspended in cold PBS by pipetting and transferred to a conical tube. The cells were pelleted, resuspended in PBS and transferred to a 1.7 mL microfuge tube before pelleting a second time. The pelleted cells were lysed in 1% NP-40 Lysis Buffer containing 1x Complete Protease Inhibitor (Roche) and 0.5 units/μL RNase inhibitor (RNasin, Promega or RnaseOUT, Invitrogen). Lysis occurred over 20 minutes on ice and was followed by centrifugation at 15,000 g for 20 minutes at 4°C to clear the insoluble fraction. The protein concentration of the lysate was quantified by DC Protein Assay (Bio-Rad). At least 1 mg of total protein was loaded onto anti-HA magnetic beads (Pierce) that had been previously blocked for 1 hr in 4% BSA with 0.5 μg/μL yeast tRNA. An aliquot of the lysate (5% of the amount used for IP) was kept for western analysis and RNA isolation. The lysate was incubated on the anti-HA beads for 1 hr with rotation at 4°C. Following binding the beads were washed twice with Low Salt Wash Buffer and twice more with High Salt Wash Buffer. The beads were resuspended in water and transferred to two fresh microfuge tubes for elution. Elution of protein was carried out by suspending the beads in reducing sample buffer (XT-Sample Buffer, Bio-Rad, with XT-sample reducing agent, Bio-Rad) and heating at 95°C for 7 minutes. Elution of RNA was performed by resuspending th beads in 200 μL of Proteinase K Buffer containing 32 units of proteinase K (NEB) and incubation for 20 minutes at 37°C. After 20 minutes an equal volume of Proteinase K Urea Buffer was added and the samples were incubated another 20 minutes at 37°C. Following incubation the RNA was extracted using low pH phenol:chloroform and precipitated by ethanol precipitation with glycogen added as a carrier. The precipitated RNA was washed with 70% ethanol, dried and resuspended in water. The RNA was DNase treated (Turbo DNase, Ambion) Isolation of RNA from the input sample was performed in the same manner as elution of the beads.

### RNA analysis by RT-qPCR

Total cDNA synthesis was performed using iScrpt Supermix per manufacturer protocol (Bio-Rad). For miRNA reverse transcription 6 pmol of the *let-7* reverse transcription primer [31] was added to the reaction containing 1X iScript Supermix and cDNA was synthesized per manufacturer protocol. Quantitative PCR was performed using iQ SYBR Green Supermix (Bio-Rad) on the CFX96 Real-Time system with Bio-Rad CFX Manager 3.0 software, with a standard 3 step PCR cycle with initial denaturation at 95°C for 3 min denaturation at 95°C 10 s, annealing at 55°C for 10 s, extension at 72°C for 30 s. Cycle threshold (C_t_) values were normalized to GAPDH, except where indicated. All qPCR and reverse transcription primers can be found in S2 Table.

### Protein analysis by western blotting

Protein samples were resolved on a 4–12% Bis-Tris precast gel (Bio-Rad). The resolved proteins were transferred using Trans-Blot SD (Bio-Rad) onto Immuno-Blot PVDF (Bio-Rad). The membrane was blocked in 5% milk in 1x PBS with 1% Tween (PBST) for a minimum of 1 hour. The following primary antibodies were used in western analysis at the given dilution: HA-HRP, 1:2000 (Santa Cruz, sc-7392); PABP, 1:1000 (Abcam, ab21060); FLAG-HRP, 1:5000 (Sigma, F1804); Beta-Actin-HRP, 1:2000 (BioLegend, 643807); GAPDH-HRP, 1:2000 (BioLegend, 649203); Nop56, 1:2000, (Bethyl, A302-721A-T); Anti-mouse IgG HRP, 1:10,000 (Cell Signaling, 7076S); Anti-Rabbit IgG HRP (Cell Signaling, 7074S). All antibodies were diluted in 5% milk in PBST and incubated with the membrane for 2 hours at room temperature or overnight at 4°C. The membrane washed with PBST prior to incubation with secondary antibody against mouse or rabbit coupled to horse-radish peroxidase (HRP) (Cell Signaling). The secondary was diluted 1:10,000 in 5% milk in PBST and allowed to incubate for 1 hour at room temperature. The membrane was washed with PBST and HRP activity was detected using SuperSignal West Pico or Dura (Thermo Scientific). The membrane was imaged by Bio-Rad Molecular Imager ChemiDoc XRS System with Image Lab software (Bio-Rad).

## Results

### General description of Urb-RIP

**Fig 1:**
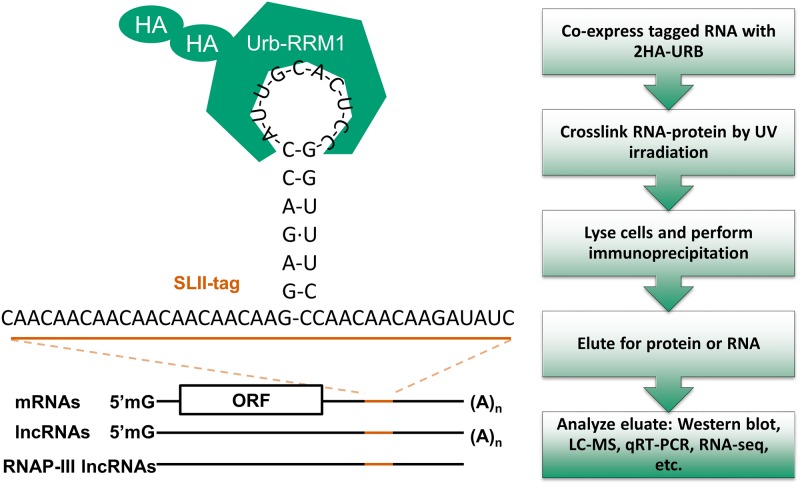
Schematic illustration of the Urb-RIP protocol and potential applications. The first step of the Urb-RIP protocol is to tag an RNA of interest with the SLII-tag, illustrated in the figure. The tagged RNA and in parallel an untagged control are coexpressed with 2HA-Urb in a cell line of interest. After a period of time the cells are UV irradiated to produce RNA-protein crosslinks. The cells are then lysed and immunoprecipitation is performed using blocked anti-HA magnetic beads. The RNA or protein is then eluted and analyzed by an appropriate method. This approach should be applicable to mRNAs and lncRNAs and amendable to a wide range of methods for eluate analysis.

**Fig 2:**
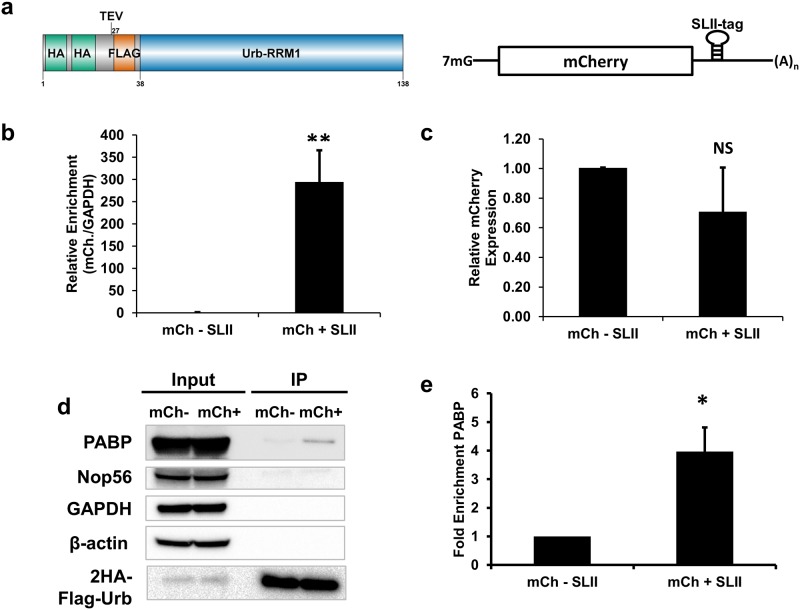
Urb-RIP enriches for mCherry-mRNA and bound PABP. **a** Schematic describing 2HA-Urb construct used for Urb-RIP and reporter, mCherry (mCh)-mRNA tagged with SLII, used to validate and optimize Urb-RIP. **b** Enrichment of mCh-mRNA by Urb-RIP as determined by qPCR. The cell line 293-2HA-Urb was transfected with a plasmid expressing mCherry-mRNA untagged or tagged with SLII. Two days after transfection the cells were UV-irradiated at 400 mJ/cm^2^ and subsequently lysed. Immunoprecipitation was performed using the Urb-RIP protocol and RNA was eluted with proteinase K treatment. qRT-PCR was performed using mCh and GAPDH primers. **c** Comparison of relative expression amounts of mCherry with (+SLII) and without (-SLII) tagging. qPCR results show a modest reduction in mCherry expression upon insertion of SLII. Relative levels of mCherry are normalized to GAPDH. **d** western blot shows enrichment of PABP following Urb-RIP of mCh. Half of the immunoprecipitate from above was eluted with sample buffer and analyzed by western blot with antibody against proteins listed. Samples labeled input represent 5% of the total sample used for Urb-RIP. **e** quantification of western blot in **d**. For panels **b** and **c** mean ± SD of three independent experiments are shown. For panel **e** mean ±SD of three independent experiments are shown. * p<0.05, ** p<0.01.

We turned to the recently “resurrected” urbilatarian homologue of the SNF/U1A/U2B family of proteins, Urb, to create Urb-RIP as a tool for pull-down of RNA of interest, Fig 1. We have tagged the RRM1 domain of Urb with two N-terminal hemmaglutanin A (HA) tags followed by a TEV protease cleavage site and a single FLAG tag, Fig 2a, 2HA-Urb. The RRM1 domain of Urb binds the U1-snRNA SLII and U2-snRNA SLIV with high affinity, 1.2 x 10^−9^ M and 1.5 x 10^−8^ M respectively [27]. This high affinity binding allows Urb-RIP to be performed with an RNA of interest bearing only a single SLII-tag. Urb is suspected to have a structure very similar to the SNF and U1A/U2B proteins for which it is a hypothetical ancestor [27]. It is expected that Urb needs a loop structure for binding, much like U1A [32]. In fact, a nearly identical variant of the Urb RRM1 domain that we are using here, Urb-V, was found to bind SLII of the U1-snRNA greater than 300 fold more efficiently than a linear RNA containing the loop sequence from SLII [33]. Hence, we were careful to maintain the SLII structure in our construct by adding unstructured ‘CAA’ repeats on either side. On the 5’ side of the stem-loop there is seven ‘CAA’ repeats while there are three on the 3’ side, Fig 1. We have also incorporated a restriction enzyme site on one side of the SLII-tag to aid in subsequent cloning. As such the total length of the engineered pull-down sequence is 60 nucleotides. The Urb-RIP procedure consists of five main steps, Fig 1. First, a SLII-tagged-RNA of interest is co-expressed with 2HA-Urb. After a period of time the cells are UV irradiated to induce RNA-protein crosslinks. Following UV irradiation cell lysis and immunoprecipitation with magnetic anti-HA matrix is performed followed by thorough washing. Prior to immunoprecipitation the anti-HA matrix is blocked with bovine serum albumin (BSA) and yeast tRNA to reduce non-specific binding. Proteinase K is used to elute the tagged RNA by degrading URB, as well as any other proteins in the IP product. The proteinase K is removed by phenol:chloroform extraction of the eluate and the RNA is precipitated using standard ethanol precipitation. The proteinase K treatment is an important step in the procedure much like in HITS-CLIP as it degrades proteins covalently bound to the RNA that could interfere with reverse transcriptase during cDNA synthesis [28]. Protein is eluted from the anti-HA matrix by adding reducing sample buffer and boiling. Finally, the eluted RNA or protein can be analyzed by a method of choice, qRT-PCR, western blot, northern blot, RNA-seq, proteomics, etc.

### Urb-RIP allows for enrichment of mRNAs

We first sought to validate Urb-RIP using a mCherry reporter containing a single SLII-tag, Fig 2a. This reporter was transfected into a stable T-REx^™^-293 cell line for the inducible expression of 2HA-Urb, we will refer to this cell line as 293-2HA-Urb. As a control, a parallel transfection was performed with a mCherry reporter lacking the SLII-tag. These reporters were used to optimize the pull-down conditions, as well as the amount of UV-irradiation and the procedure for blocking anti-HA-beads, S1, S3 and S4 Figs. The pull-down efficiency for a mCherry mRNA construct with a single SLII-tag was high for non-UV irradiated conditions (approx. 100 fold over the untagged control mRNA) and could further be improved by UV-induced RNA-protein crosslinking. We found that cross-linking with 400 mJ/cm^2^ of UV prior to lysis provided enrichment over non-irradiated samples (approximately 2 fold) for the SLII-tagged mCherry RNA, S1 Fig. Importantly we did not observe overt cleavage of RNA following UV irradiation as determined by standard analysis of rRNA integrity by denaturing agarose gel, S2 Fig. Blocking of the beads with yeast tRNA and BSA increased enrichment while pre-clearing of the lysate with protein A/G matrix improved specificity of the tagged mRNA pull-down but did not improve overall enrichment, S3 Fig. Following optimization of Urb-RIP we were able to readily obtain enrichment of our SLII-tagged mCherry reporter of ~350 fold, Fig 2b. Importantly the addition of the SLII-tag to the 3’UTR of mCherry only modestly reduced expression, Fig 2C. We found that one of the pitfalls of the Urb-RIP approach is the ability of the 2HA-Urb protein to interact with SLII of the endogenous U1-snRNA. However, we observed that blocking of matrix prior to immunoprecipitation reduced enrichment of U1-snRNA, S4 Fig.

### Urb-RIP enriches for tagged mRNA and a bound RNA binding protein

To validate Urb-RIPs ability to identify RBP bound to RNA of interest we assessed enrichment of PABP bound to a tagged mRNA. We used the mCherry reporters described above, Fig 2. These reporters were transfected into 293-2HA-Urb. Analysis of Urb-RIP using these constructs showed enrichment of mCherry-mRNA and PABP, Fig 2B, 2D and 2E. The presence of background PABP binding was not surprising as the Urb-RIP product often contains traces of non-tagged mRNAs, S3 Table. However, the tagged-RNA is efficiently immunoprecipitated and much more abundant in the Urb-RIP pull-downs than the untagged; for example the tagged RNA can be detected by qPCR approximately 8 cycles before the untagged RNA, S3 Table. While in the Urb-RIP input the untagged and tagged RNA are detected with less than a cycle difference. GAPDH can routinely be detected with a threshold cycle in the mid-thirties but is sometimes undetectable, S3 and S4 Tables. The same is true for Actin mRNA. Even highly abundant RNAs such as 7SK are much less abundant in the IP eluate than our tagged mRNA, approximately 2% of the abundance of the tagged mRNA, S3 Table. To control for potential binding of 2HA-Urb to RNAs containing a sequence similar to SLII, i.e. the loop from SLII, we used qPCR to detect binding to the TIMM50 mRNA. While TIMM50 contains a sequence identical to the loop of our SLII-tag we did not observe any enrichment upon IP, S3 Table. Importantly we did not observe binding of abundant proteins such as beta-actin or GAPDH. Also, we did not observe binding of the RBP Nop56, which binds the box C/D snoRNAs and is involved in ribosome biogenesis [34, 35].

### Urb-RIP enriches for miRNAs and Ago2

**Fig 3:**
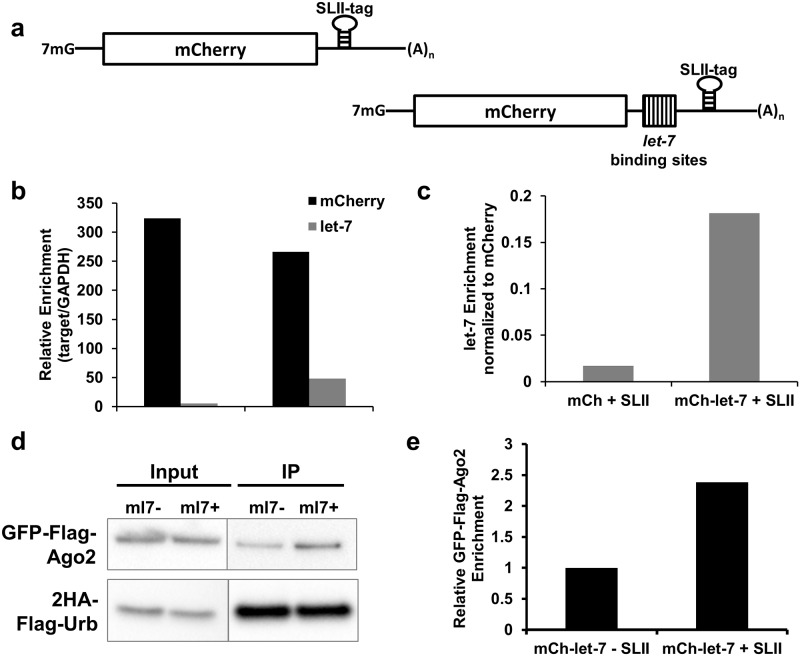
Urb-RIP shows Argonaute and miRNA binding to miRNA-targeted messages in human cells. **a** Schematic describing the *let-7* reporters used to validate Urb-RIPs ability to identify interacting miRNA. **b** Enrichment of *let-7* and mCh-mRNA by Urb-RIP as determined by qPCR. The cell line 293-2HA-Urb was transfected with plasmids expressing the constructs described in **a** as well as a plasmid for expression of GFP-FLAG-Ago2. Two days after transfection the cells were UV-irradiated at 400 mJ/cm^2^ and subsequently lysed. Immunoprecipitation was performed using the Urb-RIP protocol and RNA was eluted with proteinase K treatment. qRT-PCR was performed using mCh, *let-7* and GAPDH primers. **c** enrichment of *let-7* normalized to mCh abundance in the immunoprecipitate. **d** western blot shows enrichment of GFP-FLAG-Ago2 following Urb-RIP. The mCh-*let-7*-SLII reporters from above were co-transfected with GFP-FLAG-Ago2. Two days after transfection Urb-RIP was performed. The eluted protein as well as input was analyzed by western blot with antibody against FLAG (GFP-FLAG-Ago2 and 2HA-FLAG-Urb). Samples labeled input represent 5% of the total sample used for Urb-RIP. **e** quantification of western blot in **d,** normalized to 2HA-Urb, relative to mCh-*let-7*-SLII.

A common desire in the field of post-transcriptional regulation of gene expression is the ability to identify miRNAs and miRNA-induced silencing complexes (miRISC) that target an RNA of interest. While there exists several *in silico* tools to identify potential binding sites for a miRNA, it is often the case that their predictions produce false positives [36, 37]. An alternative approach has been to utilize HITS-CLIP or PAR-CLIP which can identify targets of a given miRNA [28, 38]. In order to identify which miRNAs are capable of binding an RNA of interest the most direct approach would be to enrich for the RNA through a pull-down. With this in mind we sought to test Urb-RIPs ability to identify a miRNA bound to an RNA of interest. We used a *let-7* reporter construct which contains multiple binding sites for *let-7* in the 3’UTR of the reporter gene [30]. The insertion of eight *let-7* sites has been shown previously to reduce expression of such reporters [30] and we have observed repression of our mCherry-*let-7* reporter by reduction in mCherry fluorescence (data not shown). We inserted the SLII-tag between the *let-7* sites and the polyadenylation signal, Fig 3A. This construct along with a control lacking the SLII-tag and the mCherry constructs described in Fig 2 were transfected into the 293-2HA-Urb cell line used above and Urb-RIP was performed two days later. Analysis of the immunoprecipitated RNAs revealed enrichment of *let-7* bound to the mCherry-*let-7* reporter, Fig 3B and 3C. We next transfected the mCherry-*let-7* reporter along with GFP-FLAG-Ago2 and performed Urb-RIP as before. In parallel we assayed the GFP-FLAG-Ago2 binding to the control reporter lacking the SLII-tag. Analysis of the Urb-RIP product by western blot revealed modest enrichment of GFP-FLAG-Ago2 in the sample containing the SLII-tagged mCherry-*let-7* reporter, Fig 3D and 3E. As seen with PABP in Fig 2 there was GFP-FLAG-Ago2 in the control sample. This can be attributed to overexpression of tagged-Ago2 construct and the presence of trace amounts of various RNA species in the control pull-down as described above.

### Urb-RIP confirms binding of PABP to the RNAPIII lncRNA BC200

**Fig 4:**
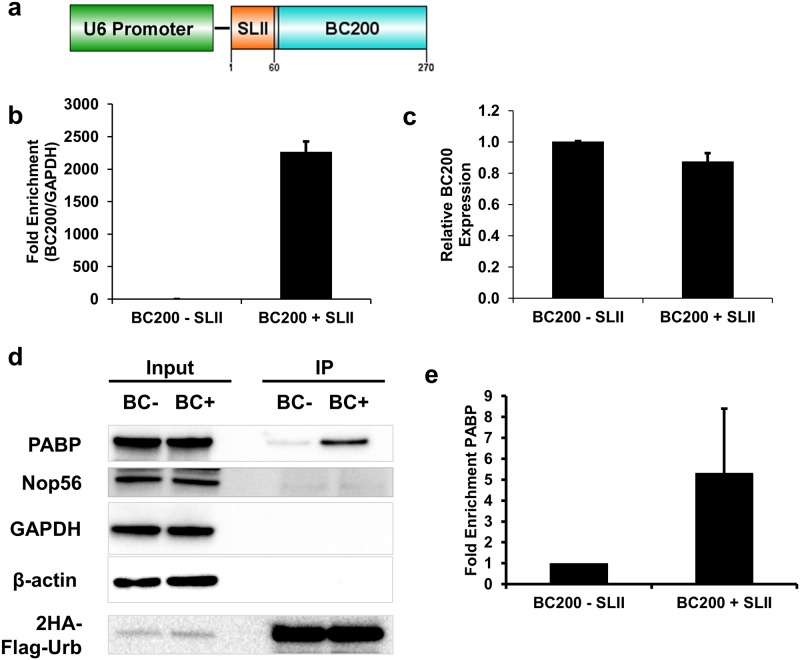
Urb-RIP confirms binding of PABP to BC200. **a** Schematic describing the BC200 construct used for pull-down by Urb-RIP. **b** Enrichment of BC200+SLII by Urb-RIP as determined by qPCR. A stable HEK-293 cell line for the inducible expression of 2HA-Urb was transfected with plasmids expressing the constructs described in **a** Two days after transfection the cells were UV-irradiated at 400 mJ/cm^2^ and subsequently lysed. Immunoprecipitation was performed using the Urb-RIP protocol and RNA was eluted with proteinase K treatment. qRT-PCR was performed using BC200 and GAPDH primers. **c** Comparison of relative expression amounts of BC200 with (+SLII) and without (-SLII) tagging. qPCR results show no change in BC200 expression upon insertion of SLII. Relative levels of BC200 are normalized to GAPDH. **d** Western blot shows enrichment of PABP following Urb-RIP of BC200+SLII. Half of the immunoprecipitate from above was eluted with sample buffer and analyzed by western blot with antibody against proteins listed. Samples labeled input represent 5% of the total sample used for Urb-RIP. **e** Analysis of western blot in **d**, normalized to 2HA-Urb, relative to mCh-SLII. For panels **b** and **c** mean ± SD of two independent experiments are shown. For panel **e** mean ±SD of three independent experiments are shown. * p<0.05.

In order to show the adaptability of Urb-RIP for other RNA transcripts we used our method to identify factors bound to the lncRNA BC200. BC200 is a well described lncRNA transcribed by RNA PolIII [39–43]. The BC200 transcript contains a large A-rich element which was shown *in vitro* to interact with PABP [40–43]. We tagged the 5’ end of BC200 with SLII and expressed it using the U6 promoter in a 2HA-Urb stable cell line as described in Fig 4A. The use of a single SLII-tag allows us to add a relatively short sequence to the natural BC200 transcript, Fig 4A, in comparison with tagging BC200 with MS2 hairpins which would approximately double or triple the length of the BC200 transcript if tagged with 12 or 24 MS2 hairpins as is common. We transfected our SLII-tagged BC200 construct as well as untagged control in parallel into the 293-2HA-Urb cell-line used previously. Urb-RIP with SLII-tagged and control BC200 expressing cells was performed two days after transfection. Analysis of the pull-down efficiency showed substantial enrichment of BC200 lncRNA, which was readily more than 2000 fold, Fig 4B and S5 Fig. The addition of the SLII-tag had no effect on BC200 expression, Fig 4C. Analysis of the immunoprecipitated RNA by bioanalyzer showed a prominent peak for BC200 in the BC200+SLII pulldown, this peak was absent from the control pulldown, S6 Fig. Furthermore, analysis of non-target RNAs by qPCR showed little binding during the pull-down of BC200, S5 Table, consistent with the results of mCherry pull-down, S3 Table. We could also confirm binding of PABP to BC200 by western blot analysis of immunoprecipitated tagged-BC200, Fig 4D and 4E. As such we could show that Urb-RIP method can be used equally well for untranslated lncRNA that may act in post-transcriptional control of gene expression.

## Discussion

We have presented Urb-RIP, an adaptable and efficient approach to affinity purify specific RNAs and to identify interacting RNAs, RBPs or RNPs. Our method uses a novel affinity tag for RNA affinity purification. We utilize the RRM domain of a recently “resurrected” snRNA-binding protein, Urb [27]. Urb-RIP takes advantage of the high affinity binding of the RRM1 domain of Urb to SLII of the U1-snRNA to affinity purify an RNA of interest using a single stem-loop tag. By epitope tagging the RRM1 domain of Urb making 2HA-Urb we can affinity purify any RNA of interest containing the SLII-tag with anti-HA matrix in a single purification step with higher efficiency than most of the current methods. In order to improve the effectiveness of this technique we have incorporated UV induced crosslinking. Much like CLIP or HITS-CLIP, the UV crosslinking used in Urb-RIP helps to stabilize RNA-protein complexes [29, 44]. While UV crosslinking is notoriously inefficient, between 1–5% [44], we have opted to include it in this protocol in order to help maintain interactions with more transient or weakly interacting RBPs. It is possible to perform Urb-RIP without UV crosslinking however we have found that it is slightly less efficient than with crosslinking, S1 Fig. We have shown that Urb-RIP can provide enrichment of RBPs and miRNAs bound to immunoprecipitated RNAs.

This method provides many advantages over the commonly used MS2 system for RNA purification. Urb-RIP requires only one SLII-tag there by limiting the mass added to the RNA of interest. The single tag also makes cloning much easier as the tag can be synthesized using a single DNA oligonucleotide and its complement and simply ligated into a plasmid of interest or added to the template sequence of an RNA of interest through PCR. We have not observed any aggregation of yellow fluorescent protein tagged 2HA-Urb or negative effects on cellular homeostasis upon continuous expression of 2HA-Urb in stable cell lines (data not shown). This gives Urb-RIP an advantage over other RNA pull down methods. Aggregation of the MS2 protein is a common problem and requires tight control of expression in order to be mitigated [4, 45]. While mutations in MS2 coat proteins may reduce the oligomerization pattern of the protein [8, 46], requirement of the multiple binding loops still increases possibility for aggregation and reduction in the immunoprecipitation of active RNP complexes on targeted RNA transcripts.

Urb-RIP proved capable of enrichment of tagged mRNAs and lncRNAs from cell lysates as well as for their *trans* regulators: ncRNAs, RBPs and RNPs, Figs 2, 3 and 4. Urb-RIP method proved capable of enriching for a miRNA and Argonaute, miRISC component, bound to an RNA of interest (Fig 3A and 3B). We used a reporter for the miRNA let-7 to confirm the ability of Urb-RIP to identify interacting miRNAs by qPCR. As such the ability of Urb-RIP to identify miRNAs bound to RNA of interest could be a very valuable tool to many researchers.

Using our method we were able to confirm binding of PABP to the PolIII *in vivo* transcribed human lncRNA BC200. We showed that human BC200 lncRNA can be efficiently immunoprecipitated using our Urb-RIP method, Fig 4. Further western blot analysis of immunoprecipatated material bound to tagged-BC200 showed subtle and reproducible enrichment of PABP. The interaction of PABP to an internal tract of adenosines in human BC200 and mouse BC1 lncRNAs has been shown previously *in vitro* by either electrophoretic mobility shift assay (EMSA) or by immunoprecipitation of PABP bound RNAs from cells transfected with *in vitro* transcribed BC200 [40–43]. By using Urb-RIP method we were able to show, for the first time, PABP and BC200 interaction by pulling-down the lncRNA.

In addition, our Urb-RIP method has been recently coupled with mass-spectrometry to identify RBPs bound to the H/ACA snoRNA ACA11. These analyses confirmed previous results [25] and revealed novel potential interactors of ACA11 snoRNA (N Mahanaj, S Liu and M Tomasson, manuscript in preparation).

An important consideration when tagging an RNA with the SLII-tag is the location the tag is to be inserted. Here we have inserted the tag into the 3’UTR of mRNAs and the 5’ end of the lncRNA BC200. For lncRNAs we suspect the tag could be placed at either end of transcript. It would likely be best to avoid the middle as to not perturb the structure of the RNA. For mRNAs the tag should be placed in the 3’UTR. Inserting the tag into the 5’UTR or coding sequence will likely lead to displacement of 2HA-Urb from the message by the translation machinery, or stalling of the ribosome or pre-initiation complex. In all cases the ideal location of the tag may need to be empirically determined.

One of the pitfalls of the Urb-RIP approach is the ability of the Urb-RRM1 domain to interact with endogenous U1-snRNA. However, this binding has not prevented us from identifying RBPs and RNAs bound to Urb-RIP purified mRNAs and lncRNAs. It may be possible to mitigate this issue by pre-clearing the lysate with an antibody against a U1-snRNP factor. Additionally, mutants of Urb RRM1 domain, which show similar or higher affinity to SLII hairpin [27], can be used for further improvement of the method. An additional pitfall of our method or any other RBP-mediated RNA-pulldown, such as pulldown with MS2, is that the RNA of interest is exogenous and in many cases overexpressed. This along with overexpression of the RBP used for pulldown, be it MS2 or 2HA-Urb, should be considered when designing the experiment.

Taken together our results show that Urb-RIP provides an adaptable and efficient approach for pull down of RNA of interest and their interacting proteins and ncRNAs. We predict Urb-RIP will work efficiently in most cell lines and can be coupled with many techniques for the analysis of interacting proteins and ncRNAs. Urb-RIP has the potential to become a useful tool in the study of post-transcriptional regulation of mRNA and the function of lncRNAs.

## Supporting Information

S1 Fig Optimization of pulldown protocol. A stable HEK-293 cell line for the inducible expression of 2HA-Urb was transfected with a plasmid expressing mCherry-mRNA untagged or tagged with SLII. Two days after transfection the cells were UV-irradiated at doses shown and subsequently lysed. Immunoprecipitation was performed using the Urb-RIP protocol and RNA was eluted with proteinase K treatment. qRT-PCR was performed using mChery and GAPDH primers. Pulldown efficiency was quantified by qRT-PCR analysis of enrichment of mCherry+SLII relative to mCherry, the abundance of both messages was normalized to GAPDH.
(TIF)

S2 Fig RNA integrity after UV irradiation. RNA was isolated from control or UV irradiated (400 mJ/cm2) 293-2HA-Urb cells using the Qiagen RNeasy Kit per manufacturer’s protocol. Two micrograms of RNA was mixed with 3 μL of 10x MOPS buffer, 6 μL of formaldehyde and formamide to 30 μL prior to denaturation at 80°C for 15 minutes. The RNA was cooled on ice and 2x RNA Loading Dye was added (10mM EDTA, 50% glycerol v/v, 0.25% bromophenol blue and xylene cyanol) along with ethidium bromide. The samples were loaded on a 1.2% denaturing agarose gel, resolved and the gel was imaged.
(TIF)

S3 Fig Optimization of blocking and preclearing. A stable HEK-293 cell line for the inducible expression of 2HA-Urb was transfected with a plasmid expressing mCherry-mRNA untagged or tagged with SLII. Two days after transfection the cells were UV-irradiated at 400 mJ/cm^2^ and subsequently lysed. The lysate was loaded onto untreated beads or beads blocked with 300 μL of 4% BSA, 0.5 μg/mL yeast tRNA in TBST. For one sample the lysate was cleared by incubation with Protein A/G beads for 1 hour prior to loading on the blocked beads. Following binding the beads were processed following the Urb-RIP protocol and RNA was eluted with proteinase K treatment. qRT-PCR was performed using mChery and GAPDH primers. Pulldown efficiency was quantified by qRT-PCR analysis of enrichment of mCherry+SLII relative to mCherry, the abundance of both messages was normalized to GAPDH.
(TIF)

S4 Fig Analysis of U1-snRNA binding during Urb-RIP. A stable HEK-293 cell line for the inducible expression of 2HA-Urb was transfected with a plasmid expressing mCherry-mRNA untagged or tagged with SLII. Two days after transfection the cells were UV-irradiated at 400 mJ/cm^2^ and subsequently lysed. The lysate was loaded onto untreated beads or beads blocked with 300 μL of 4% BSA, 0.5 μg/mL yeast tRNA in TBST. For one sample the lysate was cleared by incubation with Protein A/G beads for 1 hour prior to loading on the blocked beads. Following binding the beads were processed following the Urb-RIP protocol and RNA was eluted with proteinase K treatment. qRT-PCR was performed using mChery, U1-snRNA and GAPDH primers. **a** Enrichment of U1-snRNA relative to the input abundance was determined by qRT-PCR, normalized to GAPDH. **b** Abundance of U1-snRNA in the immunoprecipitate relative to mCherry was determined by qRT-PCR, normalized to GAPDH.
(TIF)

S5 Fig Reproducibility of Urb-RIP. Enrichment of BC200+SLII by Urb-RIP as determined by qPCR. A stable HEK-293 cell line for the inducible expression of 2HA-Urb was transfected with plasmids expressing the constructs described in Fig 4a Two days after transfection the cells were UV-irradiated at 400 mJ/cm^2^ and subsequently lysed. Immunoprecipitation was performed using the Urb-RIP protocol and RNA was eluted with proteinase K treatment. qRT-PCR was performed using BC200 and GAPDH primers. **a** and **b** Enrichment of BC200 by qRT-PCR, normalized to GAPDH, from two independent experiments. **c** and **d** Western blot analysis of PABP and 2HA-Urb abundance in the Urb-RIP product and input. Samples labeled input represent 5% of the total sample used for Urb-RIP.
(TIF)

S6 Fig Analysis of RNA Pulldown by Bioanalyzer. A stable HEK-293 cell line for the inducible expression of 2HA-Urb was transfected with plasmids expressing the constructs described in Fig 4a Two days after transfection the cells were UV-irradiated at 400 mJ/cm^2^ and subsequently lysed. Immunoprecipitation was performed using the Urb-RIP protocol and RNA was eluted with proteinase K treatment. The eluted RNA as well as RNA from the isolated from the Urb-RIP input was analyzed by Agilent 2100 Bioanalyzer. The analysis for the input samples **a** and **b** are shown as well as the IP eluate **c** and **d**. The analysis of the IP eluate shows a strong peak for BC200 in the pull-down of BC200+SLII, d, this peak was absent in the control pulldown, c. There is a peak for the U1 and U2-snRNA in both IP eluates.
(TIF)

S1 Table Cloning primers and oligonucleotides. (DOCX)

S2 Table qPCR and Reverse Transcription Primers. (DOCX)

S3 Table Pulldown of Non-target RNAs in mCherry Pulldown. (DOCX)

S4 Table Enrichment of mCherry mRNA by Urb-RIP. (DOCX)

S5 Table Pulldown of Non-target RNAs in BC200 Pulldown. (DOCX)

S1 Text Detailed Urb-RIP Protocol. (DOC)
