# Supplementary figures and images for: Urb-RIP – An Adaptable and Efficient Approach for Immunoprecipitation of RNAs and Associated RNAs/Proteins
Source: PLoS One. 2016 Dec 8;11(12):e0167877. doi: 10.1371/journal.pone.0167877 (PMC5145212; doi:10.1371/journal.pone.0167877)

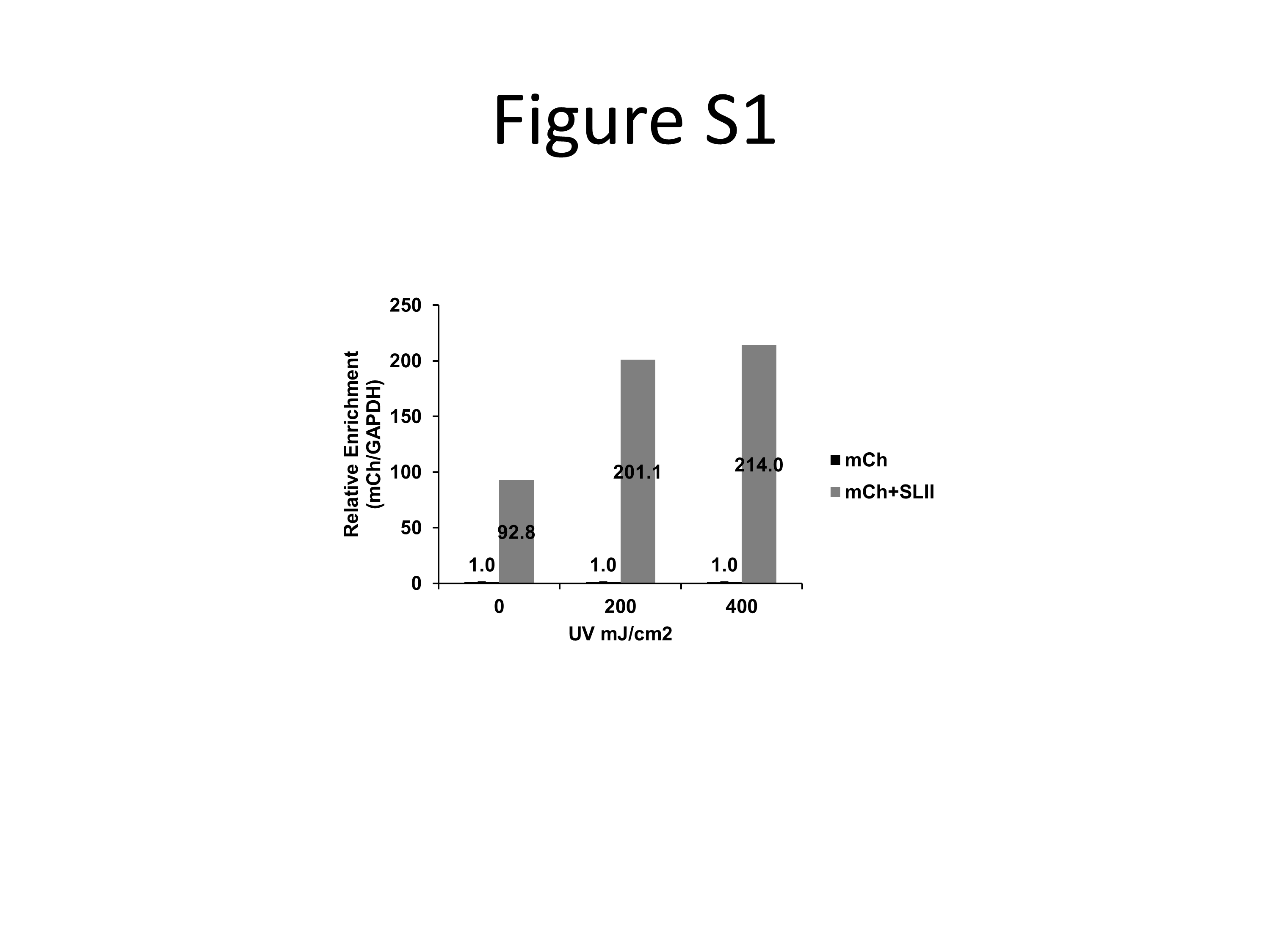

Supplement: S1 Fig — A stable HEK-293 cell line for the inducible expression of 2HA-Urb was transfected with a plasmid expressing mCherry-mRNA untagged or tagged with SLII. Two days after transfection the cells were UV-irradiated at doses shown and subsequently lysed. Immunoprecipitation was performed using the Urb-RIP protocol and RNA was eluted with proteinase K treatment. qRT-PCR was performed using mChery and GAPDH primers. Pulldown efficiency was quantified by qRT-PCR analysis of enrichment of mCherry+SLII relative to mCherry, the abundance of both messages was normalized to GAPDH. (TIF) [file pone.0167877.s001.TIF]

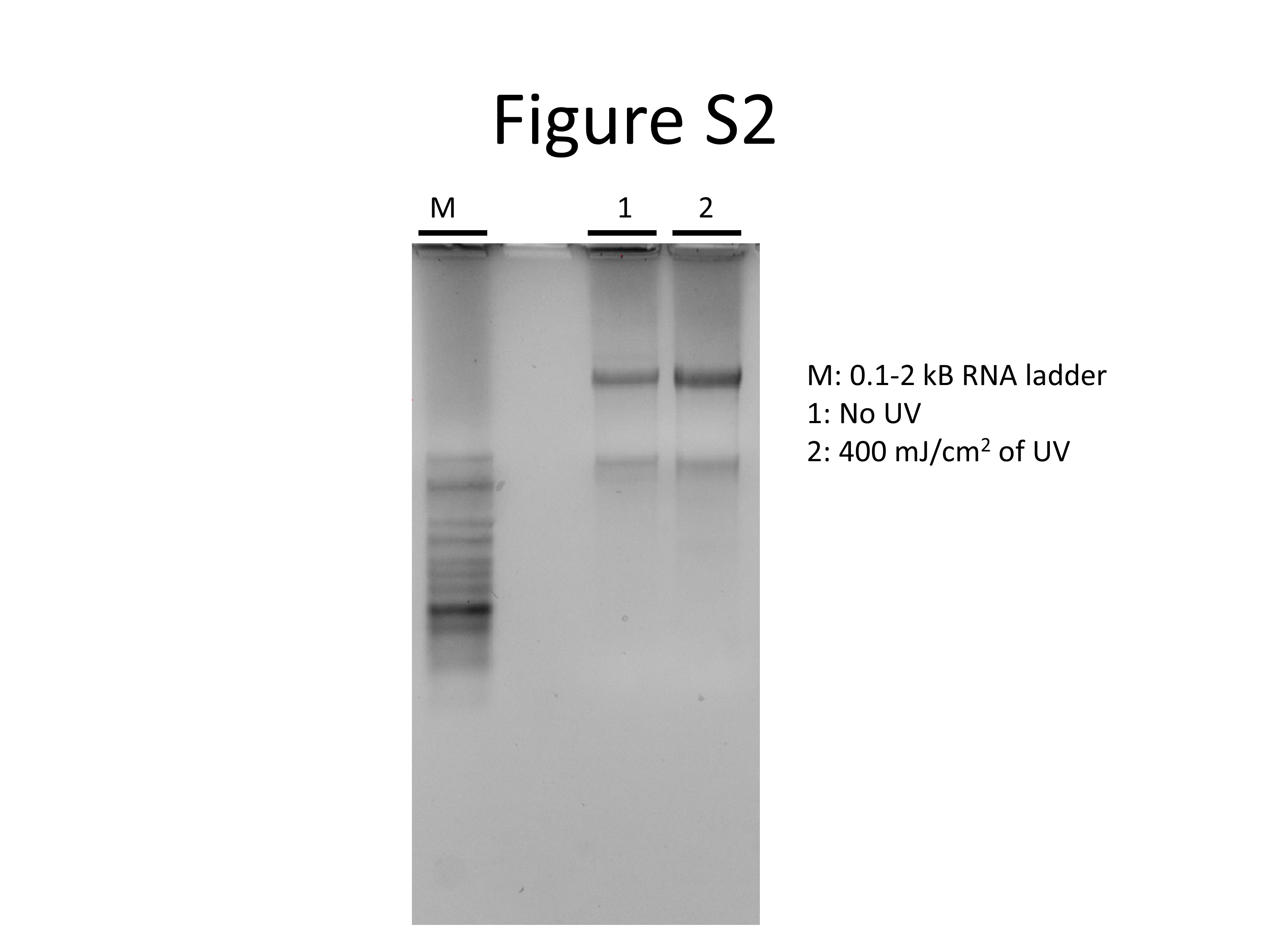

Supplement: S2 Fig — RNA was isolated from control or UV irradiated (400 mJ/cm2) 293-2HA-Urb cells using the Qiagen RNeasy Kit per manufacturer’s protocol. Two micrograms of RNA was mixed with 3 μL of 10x MOPS buffer, 6 μL of formaldehyde and formamide to 30 μL prior to denaturation at 80°C for 15 minutes. The RNA was cooled on ice and 2x RNA Loading Dye was added (10mM EDTA, 50% glycerol v/v, 0.25% bromophenol blue and xylene cyanol) along with ethidium bromide. The samples were loaded on a 1.2% denaturing agarose gel, resolved and the gel was imaged. (TIF) [file pone.0167877.s002.TIF]

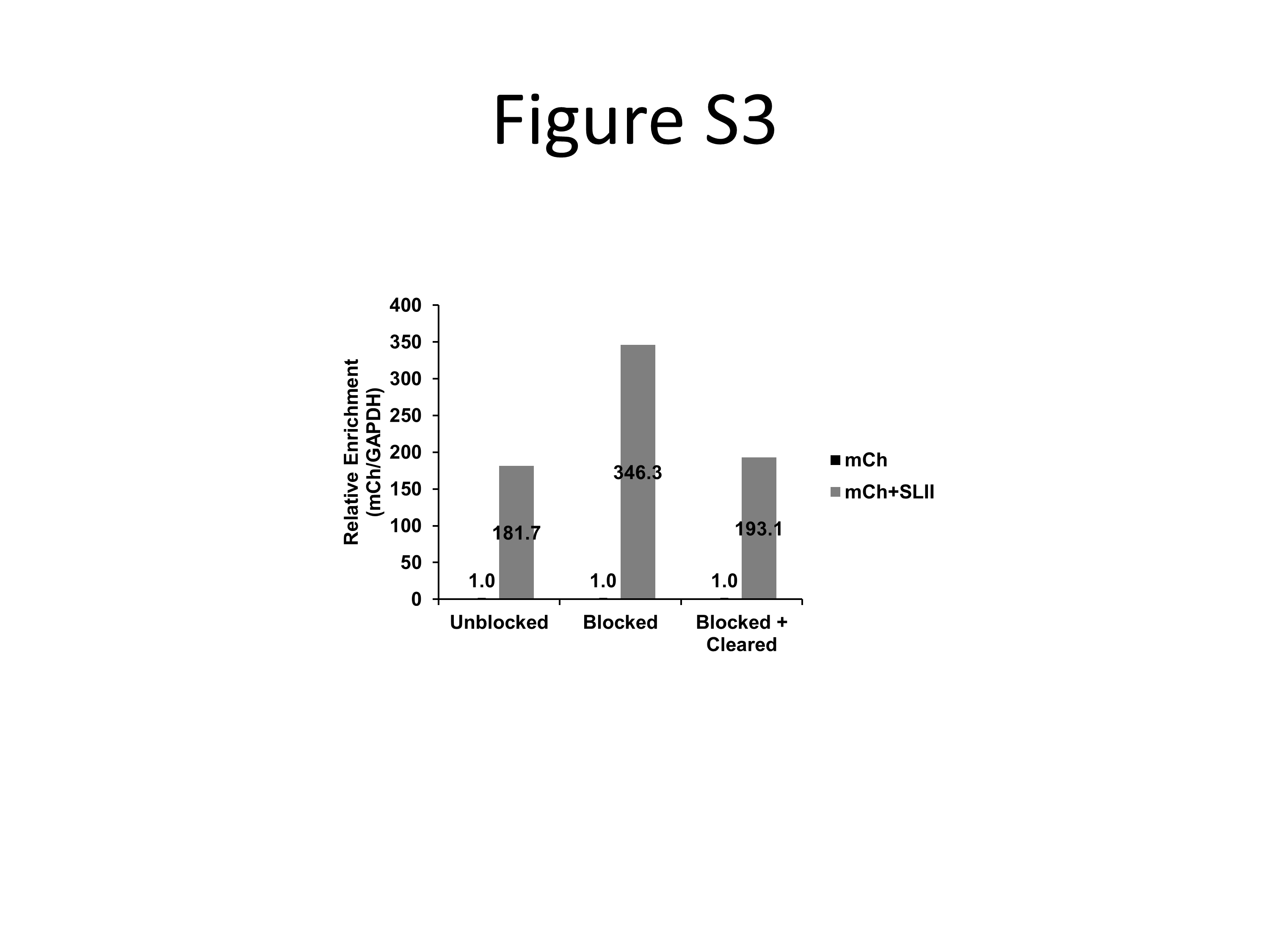

Supplement: S3 Fig — A stable HEK-293 cell line for the inducible expression of 2HA-Urb was transfected with a plasmid expressing mCherry-mRNA untagged or tagged with SLII. Two days after transfection the cells were UV-irradiated at 400 mJ/cm2 and subsequently lysed. The lysate was loaded onto untreated beads or beads blocked with 300 μL of 4% BSA, 0.5 μg/mL yeast tRNA in TBST. For one sample the lysate was cleared by incubation with Protein A/G beads for 1 hour prior to loading on the blocked beads. Following binding the beads were processed following the Urb-RIP protocol and RNA was eluted with proteinase K treatment. qRT-PCR was performed using mChery and GAPDH primers. Pulldown efficiency was quantified by qRT-PCR analysis of enrichment of mCherry+SLII relative to mCherry, the abundance of both messages was normalized to GAPDH. (TIF) [file pone.0167877.s003.TIF]

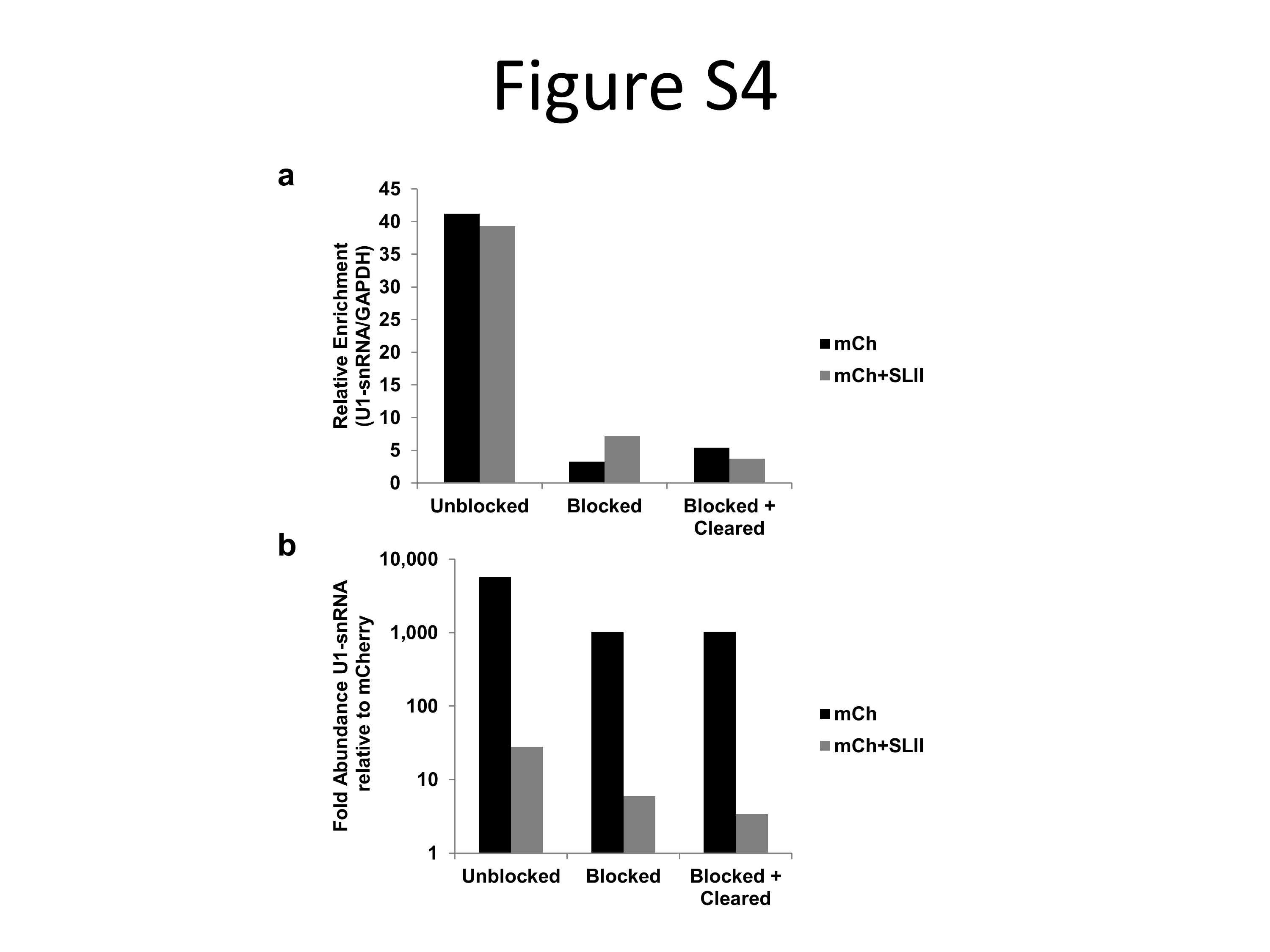

Supplement: S4 Fig — A stable HEK-293 cell line for the inducible expression of 2HA-Urb was transfected with a plasmid expressing mCherry-mRNA untagged or tagged with SLII. Two days after transfection the cells were UV-irradiated at 400 mJ/cm2 and subsequently lysed. The lysate was loaded onto untreated beads or beads blocked with 300 μL of 4% BSA, 0.5 μg/mL yeast tRNA in TBST. For one sample the lysate was cleared by incubation with Protein A/G beads for 1 hour prior to loading on the blocked beads. Following binding the beads were processed following the Urb-RIP protocol and RNA was eluted with proteinase K treatment. qRT-PCR was performed using mChery, U1-snRNA and GAPDH primers. a Enrichment of U1-snRNA relative to the input abundance was determined by qRT-PCR, normalized to GAPDH. b Abundance of U1-snRNA in the immunoprecipitate relative to mCherry was determined by qRT-PCR, normalized to GAPDH. (TIF) [file pone.0167877.s004.TIF]

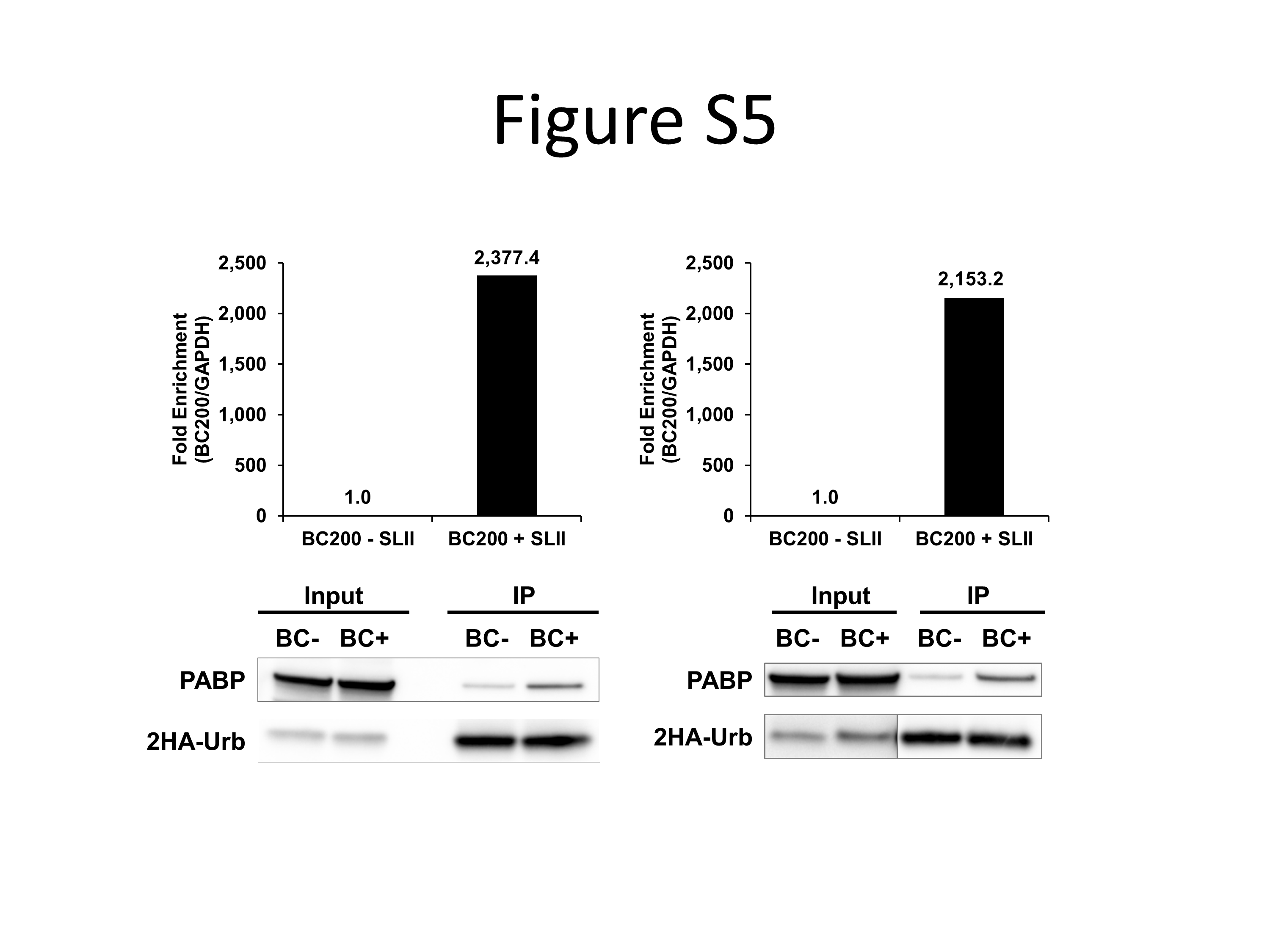

Supplement: S5 Fig — Enrichment of BC200+SLII by Urb-RIP as determined by qPCR. A stable HEK-293 cell line for the inducible expression of 2HA-Urb was transfected with plasmids expressing the constructs described in Fig 4a Two days after transfection the cells were UV-irradiated at 400 mJ/cm2 and subsequently lysed. Immunoprecipitation was performed using the Urb-RIP protocol and RNA was eluted with proteinase K treatment. qRT-PCR was performed using BC200 and GAPDH primers. a and b Enrichment of BC200 by qRT-PCR, normalized to GAPDH, from two independent experiments. c and d Western blot analysis of PABP and 2HA-Urb abundance in the Urb-RIP product and input. Samples labeled input represent 5% of the total sample used for Urb-RIP. (TIF) [file pone.0167877.s005.TIF]

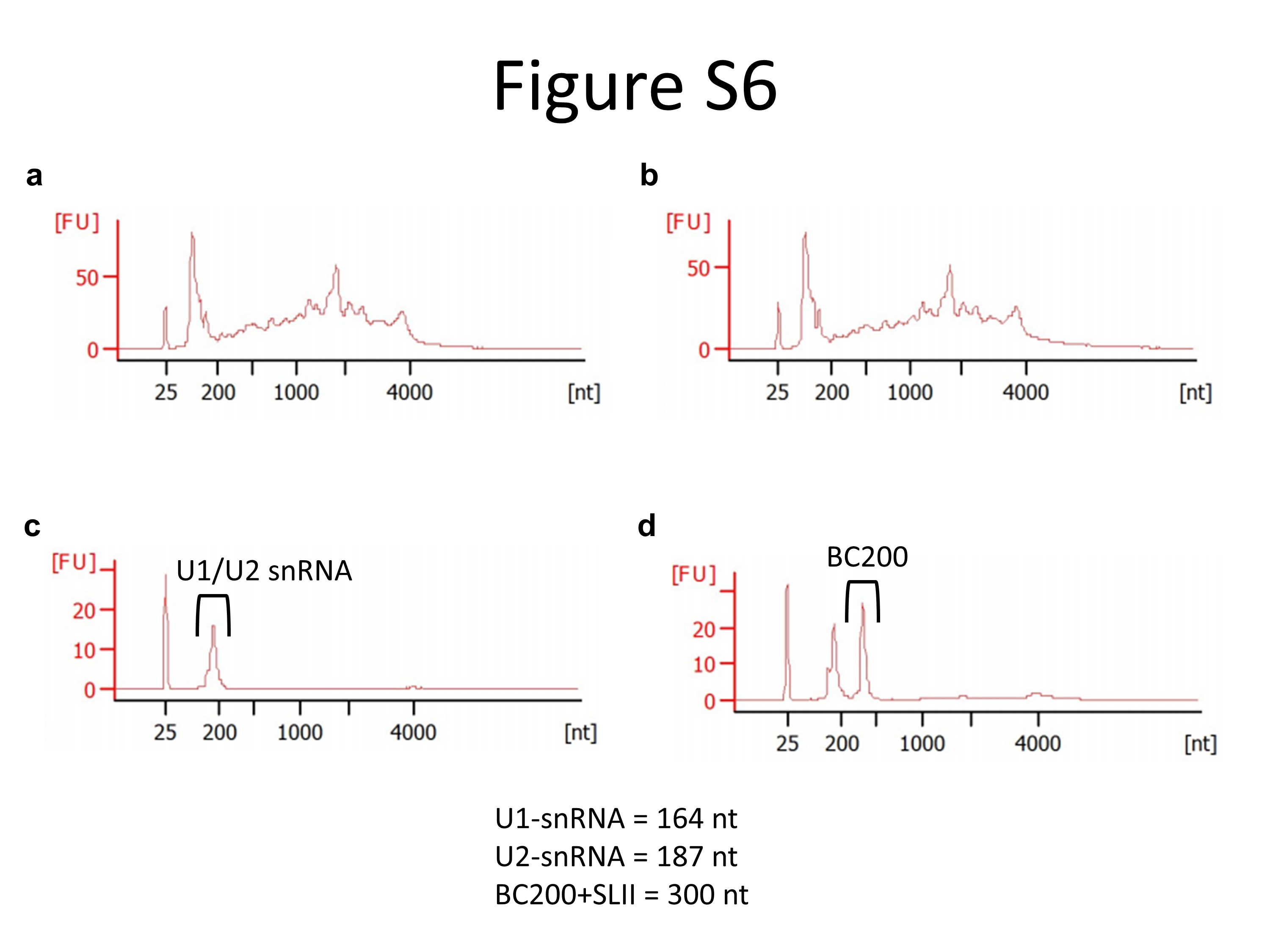

Supplement: S6 Fig — A stable HEK-293 cell line for the inducible expression of 2HA-Urb was transfected with plasmids expressing the constructs described in Fig 4a Two days after transfection the cells were UV-irradiated at 400 mJ/cm2 and subsequently lysed. Immunoprecipitation was performed using the Urb-RIP protocol and RNA was eluted with proteinase K treatment. The eluted RNA as well as RNA from the isolated from the Urb-RIP input was analyzed by Agilent 2100 Bioanalyzer. The analysis for the input samples a and b are shown as well as the IP eluate c and d. The analysis of the IP eluate shows a strong peak for BC200 in the pull-down of BC200+SLII, d, this peak was absent in the control pulldown, c. There is a peak for the U1 and U2-snRNA in both IP eluates. (TIF) [file pone.0167877.s006.TIF]
