# Supplementary material for: Urb-RIP – An Adaptable and Efficient Approach for Immunoprecipitation of RNAs and Associated RNAs/Proteins
Source: PLoS One. 2016 Dec 8;11(12):e0167877. doi: 10.1371/journal.pone.0167877 (PMC5145212; doi:10.1371/journal.pone.0167877)
Supplement: S1 Table — (DOCX) [file pone.0167877.s007.docx]

| **Supplemental Table S1: Cloning primers and oligonucleotides** | |
| --- | --- |
| Primer/Oligo Name | Sequence (5’->3’) |
| 2HA-RRM1-URB Forward 1 | ATGTATCCGTATGATGTGCCGGATTATGCGGCGGCGTATCCGTATGATGTGCCGGATTATGCGGAAAACCTGTATTTTCAGGGCGACATCCGCCCGAACCACACG |
| 2HA-RRM1-URB Forward 2 | CACCATGTATCCGTATGATGTGCC |
| 2HA-RRM1-URB Reverse | CATTAGCGTTCCACAAAGGTGC |
| 2HA-URB NarI Mut. Forward | GCGGAAAACCTGTATTTTCAGGGCGCCGACATCCGCCCGAACC |
| 2HA-URB NarI Mut. Reverse | GGTTCGGGCGGATGTCGGCGCCCTGAAAATACAGGTTTTCCGC |
| Flag Oligo 1 | CGATTACAAGGACGATGACGATAAGGG |
| Flag Oligo 2 | CGCCCTTATCGTCATCGTCCTTGTAAT |
| SLII Tag SacII Oligo 1 | GGCAACAACAACAACAACAACAAGGAGACCATTGCACTCCGGTTTCCCAACAACAAGATATCCCGC |
| SLII Tag SacII Oligo 2 | GGGATATCTTGTTGTTGGGAAACCGGAGTGCAATGGTCTCCTTGTTGTTGTTGTTGTTGTTGCCGC |
| BC200 F | CTAGACTAGTAAAGGCCGGGCGCGGTGGCTCAC |
| BC200 R (with terminator) | CTAGTCTAGAAAAAAAAGGGGGGGGGGGGTTGTTG |
| SLII Tag SpeI Oligo 1 | AATTCCAACAACAACAACAACAACAAGGAGACCATTGCACTCCGGTTTCCCAACAACAAGATATCG |
| SLII Tag SpeI Oligo 2 | TTAAGCTATAGAACAACAACCCTTTGGCCTCACGTTACCAGAGGAACAACAACAACAACAACAACC |
| CMV Deletion Oligo 1 | CCGAGGATATCCGAGA |
| CMV Deletion Oligo 2 | CTAGTCTCGGATATCCTCGGAGCT |
| pAWH *let-7* sites F | GCAGTAATTCTAGGCGATCGC |
| pAWH *let-7* sites R | CCGCTGGCCGCCTGCAGAA |
| EGFP Forward | CACCATGGGCGACTACAAGGATCACGACGGCG |
| EGFP Reverse-overlap | GCGGGGCCGGCTCCCGAGTAGGATCCGGCAGCTGCCTTGTACAGCTCGTCC |
| Ago2 Forward-overlap | GCTGTACAAGGCAGCTGCCGGATCCTACTCGGGAGCCGGCCCCGCACTTGCACC |
| Ago2 Reverse | GTAGCGGCCGCTCAAGCAAAGTACATGGTGCGCAGAGTGTCTTGG |
