# Supplementary material for: Urb-RIP – An Adaptable and Efficient Approach for Immunoprecipitation of RNAs and Associated RNAs/Proteins
Source: PLoS One. 2016 Dec 8;11(12):e0167877. doi: 10.1371/journal.pone.0167877 (PMC5145212; doi:10.1371/journal.pone.0167877)
Supplement: S2 Table — (DOCX) [file pone.0167877.s008.docx]

| **Supplemental Table S2: qPCR and Reverse Transcription Primers** | |
| --- | --- |
| Primer/Oligo Name | Sequence (5’->3’) |
| mCherry qRT Forward | CAAGGGCGAGGAGGATAACAT |
| mCherry qRT Reverse | ACATGAACTGAGGGGACAGG |
| U1-snRNA qRT Forward | CTTACCTGGCAGGGGAGATAC |
| U1-snRNA qRT Reverse | TCCGGAGTGCAATGGATAAG |
| GAPDH qRT Forward | GAGTCAACGGATTTGGTCGT |
| GAPDH qRT Reverse | GACAAGCTTCCCGTTCTCAG |
| TIMM50 qRT Forward [1] | GCGTTGGTGGTGGCGAGGTA |
| TIMM50 qRT Reverse [1] | AGCGGAGGCGGGGAAGG |
| Actin qRT Forward | AGAAAATCTGGCACCACACC |
| Actin qRT Reverse | AGAGGCGTACAGGGATAGCA |
| 7SK qRT Forward [2] | CCCCTGCTAGAACCTCCAAAC |
| 7SK qRT Reverse [2] | CACATGCAGCGCCTCATTT |
| BC200 qRT Forward | CTGGGCAATATAGCGAGACC |
| BC200 qRT Reverse | GGTTGTTGCTTTGAGGGAAG |
| *let-7* Reverse Transcription [3] | CGCATATCGCGTCATTACAGAAACTATACAA |
| *let-7* qRT Forward [3] | TCGCATATCGCGTCATTACAGA |
| *let-7* qRT Reverse [3] | GCGGAGTTGAGGTAGTAGGTTG |

1. Sankala H, Vaughan C, Wang J, Deb S, Graves PR. Upregulation of the mitochondrial transport protein, Tim50, by mutant p53 contributes to cell growth and chemoresistance. Arch Biochem Biophys. 2011;512(1):52-60. doi: 10.1016/j.abb.2011.05.005. PubMed PMID: 21621504; PubMed Central PMCID: PMCPMC3129659.

2. Galiveti CR, Rozhdestvensky TS, Brosius J, Lehrach H, Konthur Z. Application of housekeeping npcRNAs for quantitative expression analysis of human transcriptome by real-time PCR. RNA. 2010;16(2):450-61. doi: 10.1261/rna.1755810. PubMed PMID: 20040593; PubMed Central PMCID: PMCPMC2811673.

3. Wang X. A PCR-based platform for microRNA expression profiling studies. RNA. 2009;15(4):716-23. doi: 10.1261/rna.1460509. PubMed PMID: 19218553; PubMed Central PMCID: PMCPMC2661836.
