# Supplementary material for: Urb-RIP – An Adaptable and Efficient Approach for Immunoprecipitation of RNAs and Associated RNAs/Proteins
Source: PLoS One. 2016 Dec 8;11(12):e0167877. doi: 10.1371/journal.pone.0167877 (PMC5145212; doi:10.1371/journal.pone.0167877)
Supplement: S3 Table — (DOCX) [file pone.0167877.s009.docx]

| **Supplemental Table S3. Pulldown of Non-target RNAs in mCherry Pulldown** | | | | | | | |
| --- | --- | --- | --- | --- | --- | --- | --- |
|  | **Average Ct** | | | |  | |  |
|  | **Input**  **(mCh – SLII)** | **Input**  **(mCh + SLII)** | **IP**  **(mCH – SLII)** | **IP**  **(mCh + SLII)** | | **% Input**  **(mCh - IP)** | **% Input**  **(mCh + IP)** |
| **mCherry** | 23.05 | 23.66 | 35.13 | 26.93 | | 0.0231 | 10.3913 |
| **GAPDH** | 27.47 | 28.41 | N/A | N/A | | N/A | N/A |
| **Actin** | 27.72 | 28.06 | 41.01 | 43.19 | | 0.0100 | 0.0028 |
| **7SK** | 20.85 | 21.41 | 32.33 | 32.46 | | 0.0349 | 0.0472 |
| **TIMM50** | 32.00 | 32.37 | N/A | N/A | | N/A | N/A |
| **18s rRNA** | 11.72 | 12.45 | 27.79 | 25.53 | | 0.0015 | 0.0115 |
| **U1 snRNA** | 18.79 | 19.15 | 19.09 | 18.74 | | 81.4040 | 132.5872 |
| **U2 snRNA** | 21.01 | 21.28 | 24.21 | 24.09 | | 10.8633 | 14.2748 |
|  |  |  | N/A : Not detected within 45 cycles | | | | |
