# Supplementary material for: Urb-RIP – An Adaptable and Efficient Approach for Immunoprecipitation of RNAs and Associated RNAs/Proteins
Source: PLoS One. 2016 Dec 8;11(12):e0167877. doi: 10.1371/journal.pone.0167877 (PMC5145212; doi:10.1371/journal.pone.0167877)
Supplement: S4 Table — (DOCX) [file pone.0167877.s010.docx]

| **Supplemental Table S4. Enrichment of mCherry mRNA by Urb-RIP** | | | | | | | | |
| --- | --- | --- | --- | --- | --- | --- | --- | --- |
|  | **Input** | | | | **IP Eluate** | | | |
| **qPCR Target** | **mCherry** | **mCherry** | **GAPDH** | **GAPDH** | **mCherry** | **mCherry** | **GAPDH** | **GAPDH** |
| **Transfected** | **mCh-SLII** | **mCh+SLII** | **mCh-SLII** | **mCh+SLII** | **mCh-SLII** | **mCh+SLII** | **mCh-SLII** | **mCh+SLII** |
| **Trial 1** | 25.04 | 25.02 | 29.29 | 29.29 | 28.44 | 21.01 | 31.32 | 32.33 |
| **Trial 2** | 19.61 | 20.32 | 19.61 | 20.32 | 25.86 | 20.15 | 32.18 | 34.78 |
| **Trial 3** | 23.74 | 25.36 | 28.99 | 29.62 | 29.24 | 21.44 | 33.38 | 33.32 |
|  |  |  |  |  | **mCh-GAPDH** | **mCh-GAPDH** | **Enrichment** |  |
|  |  |  |  | **Trial 1** | -2.88 | -11.32 | 345.83 |  |
|  |  |  |  | **Trial 2** | -6.32 | -14.63 | 316.95 |  |
|  |  |  |  | **Trial 3** | -4.14 | -11.88 | 213.73 |  |
