# Supplementary material for: Urb-RIP – An Adaptable and Efficient Approach for Immunoprecipitation of RNAs and Associated RNAs/Proteins
Source: PLoS One. 2016 Dec 8;11(12):e0167877. doi: 10.1371/journal.pone.0167877 (PMC5145212; doi:10.1371/journal.pone.0167877)
Supplement: S5 Table — (DOCX) [file pone.0167877.s011.docx]

| **Supplemental Table S5. Pulldown of Non-target RNAs in BC200 Pulldown** | | | | | | |
| --- | --- | --- | --- | --- | --- | --- |
|  | **Average Ct** | | | |  |  |
|  | **Input (BC200 - SLII)** | **Input (BC200 + SLII)** | **IP (BC200 - SLII)** | **IP (BC200+ SLII)** | **% Input (BC200 - IP)** | **% Input (BC200 + IP)** |
| **BC200** | 14.28 | 14.08 | 26.72 | 17.48 | 0.018 | 9.433 |
| **GAPDH** | 22.73 | 22.51 | 36.12 | 40.57 | 0.009 | 0.000 |
| **Actin** | 23.42 | 23.41 | 38.69 | 35.26 | 0.003 | 0.027 |
| **7SK** | 22.34 | 22.24 | 30.21 | 30.46 | 0.428 | 0.335 |
| **TIMM50** | 26.20 | 26.44 | 35.31 | 34.51 | 0.180 | 0.372 |
| **18s rRNA** | 11.66 | 11.16 | 26.32 | 25.62 | 0.004 | 0.004 |
| **U1 snRNA** | 18.90 | 18.83 | 17.38 | 18.58 | 285.56 | 118.88 |
| **U2 snRNA** | 20.15 | 19.95 | 20.44 | 22.67 | 81.61 | 15.18 |
|  |  |  | N/A : Not detected within 45 cycles | | | |
