# Supplementary material for: Urb-RIP – An Adaptable and Efficient Approach for Immunoprecipitation of RNAs and Associated RNAs/Proteins
Source: PLoS One. 2016 Dec 8;11(12):e0167877. doi: 10.1371/journal.pone.0167877 (PMC5145212; doi:10.1371/journal.pone.0167877)
Supplement: S1 Text — (DOC) [file pone.0167877.s012.doc]

Before you begin:

- Make PK/7 M urea buffer, recipe follows protocol.

Cross Linking:

1. Remove media from cells and wash with 10 mL ice-cold PBS.
2. Add 2 mL of ice-cold PBS to the plate to keep the cells moist. Keep plate on ice until crosslinking.
3. Crosslink in Stratalinker 1800 at 400 mJ/cm2.
4. Add 8 mL of ice-cold PBS to the plate and collect cells by pipetting or scraping. Transfer suspension to a 15 mL conical tube.
5. Spin cells at 1000 g for 3 minutes at 4 °C.
6. Remove supernatant and resuspend in 1 mL of cold PBS and transfer to a 1.7 mL tube.
7. Spin cells as before.
8. Remove supernatant and estimate cell volume.

Lysis and Sample preparation.

1. Lyse cells with 3-4 volumes of 1% NP-40 lysis buffer + PI with 0.5 units/uL RNase inhibitor
   1. Lyse on ice for 20 minutes
   2. Spin at maximum speed for 20 minutes at 4 °C.
   3. Transfer supernatant to a fresh tube
2. Quantitate total protein in lysate
   1. Calculate volume of lysate needed for each sample to IP at least ~1000 µg of total protein, reserve 5-10% for input RNA and Western.
      1. Bring lysate for IP to 300 µL with lysis buffer, add RNase inhibitor to 0.5 units/µL
      2. For input RNA add 200 µL proteinase K buffer with proteinase K (**pre-incubated**). Follow procedure for RNA elution below.
      3. For western input control add sample buffer to 1x
         1. Boil for 7 minutes and store at -20 °C.

Blocking Beads

1. For each IP you will need one tube of blocked anti-HA beads
2. Add 50 uL of beads to a 1.5 mL tube add 150 uL of TBS-T, vortex
3. Separate beads with magnet and remove supernatant
4. Add 1 mL of TBS-T, mix by inversion for 1 minute, collect beads with magnetic stand and remove supernatant
5. Add 300 uL of blocking buffer (lysis buffer w/PI, 4% BSA) add 15 uL of yeast tRNA (10 mg/mL) block for 1 hr with rotation at 4 C.
6. Separate beads and remove the supernatant.
7. Wash with 300 uL of TBS-T three times, leave in last wash at 4 C until ready for binding.

Binding

1. Remove TBS-T from blocked anti-HA beads
2. Add lysate, allow to bind at 4 °C for 1 hr with rotation

Washing

1. Separate beads, remove and save supernatant
   1. Transfer 5% of supernatant (15 µL) and add sample buffer to 1x
   2. Boil for 7 minutes.
2. Wash twice with low salt wash buffer, 500 µL/wash, vortex for 10s at ~1000rpm
3. Wash twice with high-salt wash buffer, 500 µL/wash, vortex for 10s at ~1000rpm
4. Separate beads, remove the last wash and add 500 µL of pure water
5. Mix and split beads into two tubes, one for protein elution and one for RNA elution.

Elute for protein or RNA

- For protein add 1x sample buffer
  1. Boil beads for 7 minutes and transfer the supernatant to a new tube.
- For RNA add 200 µL of proteinase K buffer + proteinase K (160 µL of proteinase K buffer and 40 µL Proteinase K (NEB)). **Note**: make a mastermix of the proteinse K buffer and incubate with proteinase K for 20 minutes prior to elution to kill RNase.
  1. Incubate 20 minutes at 37 °C, 1000 rpm.
  2. Add 200 µL of PK/Urea buffer.
  3. Incubate as above.
  4. Add 400 µL of acid-phenol/chloroform, vortex and let site for 5 minutes
  5. Spin at maximum speed in cold centrifuge for 15 minutes
  6. Take aqueous phase and add 1 µL of glycogen , 1/10^th^ volume of 3 M NaOAc (pH 5.5) and 2.5 volumes of 100% ethanol.
  7. Precipitate overnight at -20 °C.
  8. Spin at maximum speed in cold centrifuge for 30 minutes
  9. Wash pellet in 1 mL of 70% ethanol
  10. Let dry for 5 minutes at RT
  11. Resuspend in 10-20 µL of water.

**Buffers:**

1% NP40 Lysis Buffer (50 mL)

1% NP40 (0.5 mL)

150 mM NaCl (1.5 mL)

50 mM Tris-HCl, pH 7.8 (2.5 mL + 45.5 mL water)

Low Salt Wash Buffer (200 mL)

1x PBS (20 mL) + (168 mL water)

0.1% SDS (1 mL)

0.5% deoxycholate (10 mL)

0.5% NP-40 (1 mL)

High Salt Wash Buffer

5x PBS (100 mL) + (88 mL water)

0.1% SDS (1 mL)

0.5% deoxycholate (10 mL)

0.5% NP-40 (1 mL)

1.25X Proteinase K Buffer

125 mM Tris-Cl pH 7.8

62.5 mM NaCl

12.5 mM EDTA

PK/7 M urea buffer (make fresh) [5 ml]

100 mM Tris-Cl pH 7.8 [500 µL 1 M Tris-HCl]

50 mM NaCl [50 µL 5 M NaCl]

10 mM EDTA [100 µL EDTA]

7 M Urea [2.1021 g Urea, 2.73 mL water]
